# Comprehensive Perspectives on Experimental Models for Parkinson’s Disease

**DOI:** 10.14336/AD.2020.0331

**Published:** 2021-02-01

**Authors:** Minjing Ke, Cheong-Meng Chong, Qi Zhu, Ke Zhang, Cui-Zan Cai, Jia-Hong Lu, Dajiang Qin, Huanxing Su

**Affiliations:** ^1^State Key Laboratory of Quality Research in Chinese Medicine, Institute of Chinese Medical Sciences, University of Macau, Macao, China; ^2^Guangzhou Regenerative Medicine and Health Guangdong Laboratory, The Fifth Affiliated Hospital of Guangzhou Medical University, Guangzhou, China; ^3^South China Institute for Stem Cell Biology and Regenerative Medicine, Guangzhou Institute of Biomedicine and Health, Chinese Academy of Sciences, Guangzhou, China.

**Keywords:** Parkinson’s disease, experimental model, transgenic animal, cellular model, cross-species model

## Abstract

Parkinson’s disease (PD) ranks second among the most common neurodegenerative diseases, characterized by progressive and selective loss of dopaminergic neurons. Various cross-species preclinical models, including cellular models and animal models, have been established through the decades to study the etiology and mechanism of the disease from cell lines to nonhuman primates. These models are aimed at developing effective therapeutic strategies for the disease. None of the current models can replicate all major pathological and clinical phenotypes of PD. Selection of the model for PD largely relies on our interest of study. In this review, we systemically summarized experimental PD models, including cellular and animal models used in preclinical studies, to understand the pathogenesis of PD. This review is intended to provide current knowledge about the application of these different PD models, with focus on their strengths and limitations with respect to their contributions to the assessment of the molecular pathobiology of PD and identification of the therapeutic strategies for the disease.

Parkinson's disease (PD) ranks second among the most common neurodegenerative diseases affecting millions of patients [1]. Selective loss of neurons in substantia nigra pars compacta (SNpc) constitutes nigrostriatal deficits observed in PD patients, such as tremor, rigidity, and akinesia, which can be substantially improved by treatment with dopamine modulators [2]. However, dopamine modulators often carry serious side effects and lose effectiveness after a period of treatment [3]. Non-motor symptoms, such as autonomic dysfunction, cognitive impairment, depression, sleep disorders, pain, and fatigue, are frequently experienced by PD patients and affect their quality of life [1]. James Parkinson described PD symptoms almost two centuries ago [4], but the etiology of PD has yet to be determined. Effective therapies for PD remain unavailable.

Theoretically, PD is characterized into two forms: sporadic PD and familial PD. Sporadic PD is mostly late-onset [5] and widely recognized as outcomes of the interactive effects of multiple genetic factors and environmental factors [6]. The pathogenesis of sporadic PD is still unclear, but its key factors include long-term exposure to toxicants, including pesticides, metals, and solvents [7]. Familial PD, which is caused by gene mutations, comprises approximately 10% of PD patients [8]. Studies on the pathology of PD have indicated that deterioration in PD is caused by the formation of α-synuclein inclusion bodies that develop into globular Lewy bodies or Lewy neurites [9]. Alpha-synuclein aggregation is not only recognized as a key event in familial PD but is also the most important component of Lewy body pathology in sporadic PD [10]. Accumulating evidence suggests that mitochondrial dysfunction, oxidative and nitrative stress, neuroinflammation, abnormal aggregation of α-synuclein, and impaired autophagy are critically involved in the progression of PD [11-14], but the etiology and pathogenesis of PD are still unknown.

**Figure 1.**
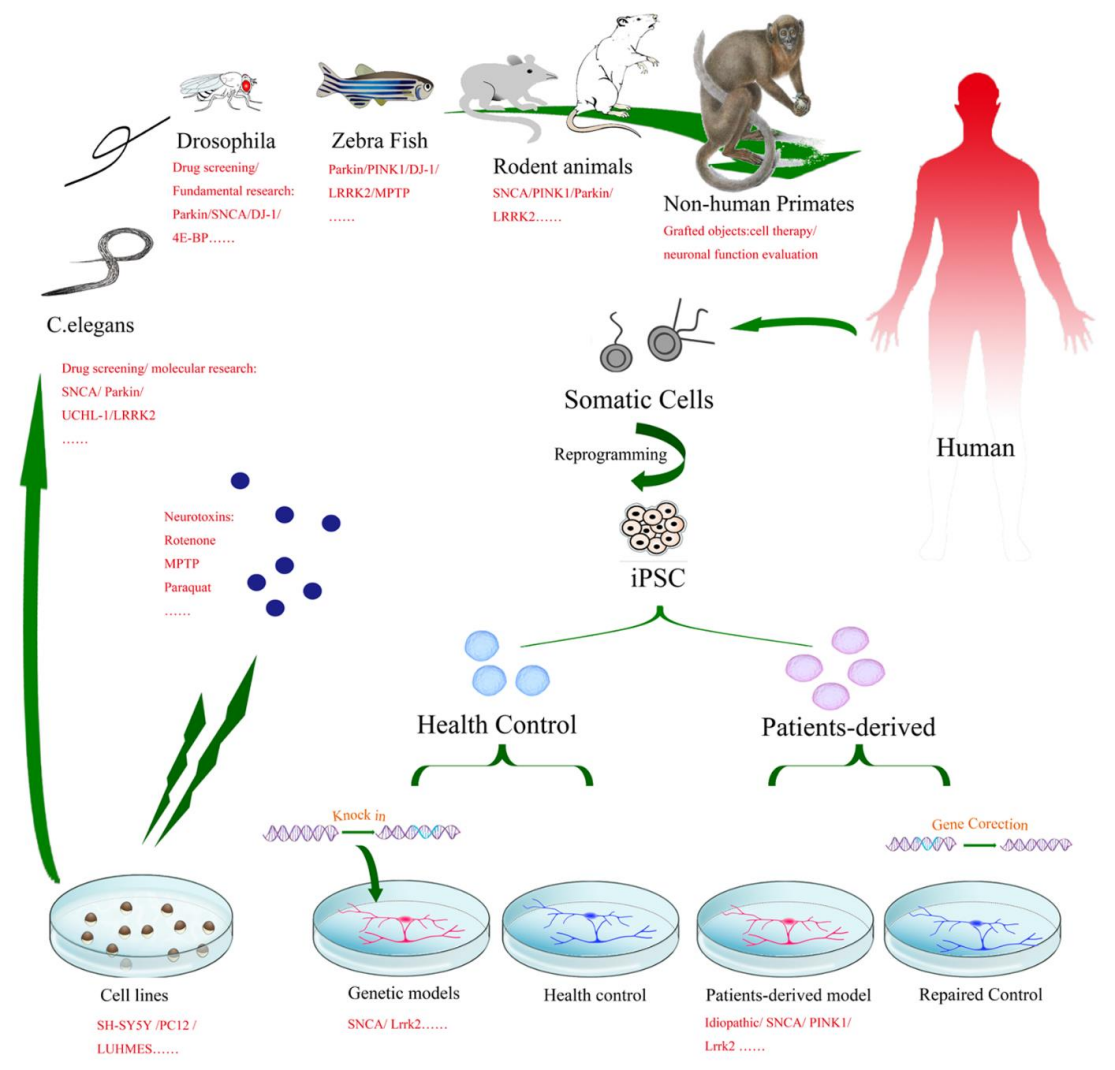
A cross-species platform for study of PD and drug test. From laboratory to clinical (bench to bedside), non-mammalian models such as cell lines, C. elegans, Drosophila and zebra fish offer an ideal and cost-effective drug screening and preliminary evaluation platform to explore hypothesized pathways or therapeutic targets. Rodent animal models, either toxin-induced or genetically established, could reproduce the lesion of DA or non-DA neurons and exhibit some of typical parkinsonian syndromes. Human iPSCs either derived from PD patients through reprogramming technology or established by gene-editing technology could provide a promising model for fundamental research and drug screening for PD. The comprised cross-species platform may accelerate the translation of laboratory research to clinical application.

Various cross-species preclinical models, including cellular models and animal models, have been established through the decades to study the etiology and mechanism of the disease from cells to nonhuman primates (NHPs), including immortal cell lines, pluripotent stem cells (PSCs), nematodes (e.g., *Caenorhabditis elegans*), fruit flies, rodent animals, and NHPs (Fig.1). These models are aimed at developing effective therapeutic strategies to slow or stop the progress of the disease. The advantages of cellular models over other models include the following: 1) they develop disease phenotypes more quickly; 2) they can more easily and reliably perform genetic or pharmacological manipulations and time-lapse imaging; 3) they require no ethical approval; and 4) they cost less. These properties of cellular models allow for large-scale testing within a short duration. In addition, specific cell types such as dopaminergic neurons can be isolated for research, which facilitates the determination of PD pathogenesis. Animal models have the advantage over cellular models of being able to recapitulate complex brain circuitry necessary to examine network dysfunction in PD. In recent decades, animal models of PD mainly consisted of two types: neurotoxin-based models and genetic models. Modeling PD is often based on the biochemical abnormalities identified in the brains of PD patients, including oxidative stress and mitochondrial dysfunction. Animal models thus provide valuable tools for identifying pathological mechanisms and modeling human diseases at distinct developmental stages *in vivo*.

In this review, we systemically summarize experimental PD models, including cellular and animal models used in preclinical studies, to understand the pathogenesis of the disease. This review aims to provide current knowledge about the use of these different PD models, with focus on their strengths and limitations with respect to their contributions to the assessment of molecular pathobiology and identification of therapeutic strategies.

## Cellular Models for PD

Cellular models fail to provide behavioral assessment and pharmacokinetics features. However, cellular models can be used to efficiently dissect a complex pathological process. Cell-based PD models have substantially contributed to the study of the underlying mechanisms and drug discovery of PD. Permanently established neuronal cell models and non-neuronal immortal cells including SHY5Y, PC12, and N2A are treated with various neurotoxins or overexpressed with PD-related genes to induce neuronal disease features in PD for the exploration of biochemical pathways and drug screening (Table 1).

### SH-SY5Y

SH-SY5Y was cloned from a bone marrow biopsy-derived cell line, SK-N-SH, and first reported in 1973 [33]. This cell line is most widely used in PD research because of its human origin and ease of maintenance. SH-SY5Y was initially characterized by its catecholaminergic (although not strictly dopaminergic) neuronal properties, expressing dopaminergic markers and presenting adrenergics in phenotypes rendering it appropriate as a model for PD [34, 35]. SH-SY5Y expresses the dopamine transporter (DAT), which is solely expressed in the central nervous system (CNS) to regulate dynamic equilibrium by uptake and storage of dopamine [36]. Although SH-SY5Y cells comprise an immortalized cell line, they still maintain their potential to differentiate into neuronal-like cells. Chemicals such as retinoic acid and cholesterol can induce differentiation of SH-SY5Y cells to express neuronal markers such as neuronal nuclei (NeuN), neuron specific enolase (NSE), growth-associated protein 43 (GAP-43), synaptophysin (SYN), microtubule-associated protein (MAP), and synaptic vesicle protein II (SV2) [37-39]. Other factors, such as estradiol (E2) and brain-derived neurotrophic factor (BDNF), exert protective effects on cell growth and morphology, synaptic vesicle recycling, and stability of neurofilaments [40, 41]. Under these conditions, the cells express cell neurites and adopt a neuron-like phenotype, which can be used as a committed model for drug screening of neurodegenerative disease.

Any of rotenone, MPTP, and 6-OHDA exerts effects by inhibiting redox reaction production complex I, contributing to a similar outcome that induces cell apoptosis. These neurotoxin-based models on SH-SY5Y are successively applied in neuroprotection research or candidate drug discovery. Neurotoxin-induced models can cause endoplasmic reticulum (ER) stress in SH-SY5Y cell lines and inhibition of AMPK/mTOR signaling [15, 16]. Many attempts regulating AMPK/mTOR pathway by overexpression of miR185 [19] or miR124 [17] in SH-SY5Y can attenuate the PD-related phenotype. SIRT3 protects a rotenone-induced PD cell model on SH-SY5Y via activation of the liver kinase B1-AMP-activated protein kinase-mTOR pathway[21]. DAT plays an important role in the neurotoxic process [42], and MPP-exhibits high affinity to DAT on dopaminergic neurons [18]; thus, MPTP-induced models are most widely used in Parkinsonism research or neuronal protective drug screening among these three neurotoxins. A novel study indicated that silencing PGC-1 alpha in MPTP-treated SH-SY5Y improved cell viability and mitochondrial function [20], presenting a potential therapeutic target in PD drug discovery.

**Table 1:** Summary of key features of immortal cell lines in PD modeling.

Cell lines	Description	Differentiated markers	Application		Ref.
			Treatments	Key Features	
SH-SY5Y	Catecholaminergic neuronal properties (although not strictly dopaminergic), presenting adrenergics in phenotypes, expressing DA neuron related markers, and being manipulated esaily	NeuN, NSE, GAP-43, SYN, MAP2 and SV2	6-OHDA	Increased LDH, loss of cell viability, and ER stress	[15, 16]
			MPP+/MPTP	Apoptosis, oxidative stress, activation of the AMPK/mTOR signaling pathway, and downregulation of PCG-1α	[17-20]
			Rotenone	Accumulation of α-synuclein, oxidative stress, and mitochondrial dysfunction	[21]
PC12	Having a closer identity to the adrenergic nerve ending	Monoamine oxidase A and Catecholamines	6-OHDA	Decreased neurites, apoptosis, mitochondrial dysfunction, upregulation of CRCX4, and ER stress	[22, 23]
			Rotenone	ER stress	
			MPP+	ER stress	
LUHMES	Capable of differentiating into DA-like neurons with various PD-associated phenotypes.	TH, α-Synuclein	Point mutations (A53T, E46K, E35K, E57K, and A30P)	Increased aggregation of α-Synuclein	[24]
			MPP+/MPTP	Increased LDH, loss of neuronal characteristics, and declined ATP production	[25]
			6-OHDA	upregulation of PD-related genes	[26]
MEs23.5	Moderately expressing TH, voltage-gated Ca2+ channels and can be long-term cultured	TH	MPP+/MPTP	Depletion of dopamine and metabolite, apoptosis, mitochondrial dysfunction, and decrease of DA neuron characteristic	[27, 28]
			6-OHDA	Ferrous iron influx in cells, and accumulation of mito-ROS	[29]
MN9D	Able to synthetize, release, and uptake dopamine independently	Not reported	6-OHDA	Apoptosis, release of cytochrome C, and decrease in p-CREB	[30]
			MPP+/MPTP	Decreased expression of Sphk2 and p-CREB, mitochondrial dysfunction, and declined ATP production	[31]
			Rotenone	Swollen mitochondria, disrupted membrane, and depletion of cellular ATP	[32]

### PC12

PC12 is a cell line established from a pheochromocytoma of the rat adrenal medulla originated from the neural crest consisting of neuroblastic and eosinophilic cells. PC12 can faithfully differentiate into neuron-like cells and perform neuronal functions in a defined medium supplemented with nerve growth factors (NGF) [43]. The morphology of differentiated PC12 is similar to that of sympathetic neurons, gradually forming sympathetic neuron-like synapses after being cultured in a defined medium with NGF. Volumes of soma waxes and synapses increasingly multiply, forming a network [44, 45]. Compared with chromaffin cells, PC12 cells have a closer identity to the adrenergic nerve ending, as revealed by the presence of monoamine oxidase A and tyramine-released pool of catecholamines [46]. Rideout et al. used a proteasome inhibitor and lactacystin to treat differentiated and undifferentiated PC12 cells. They found that the cell apoptosis rates of both cell types were dose-dependent, and α-synunclein staining was positive [47], successfully mimicking Lewy body and cell apoptosis.

Neurotoxin agents are widely used on PC12, including MPTP, rotenone, and 6-OHDA. Ryu et al. depicted the three neurotoxins in differentiated PC12 to measure ER stress and the unfolded protein response (UPR) [22]. He concluded that ER stress and UPR induced by 6-OHDA is considerably more violent than those induced by rotenone and MPTP. The ER stress response could be a convergent effect of these agents which will subsequently act on mitochondria, and oxidative stress may be responsible for inducing ER stress [22]. Resveratrol protects PC12 from 6-OHDA damage by activating the CXCR4 signaling pathway [23]. CXCR4 is a specific G-protein-coupled 7-transmembrane span receptor that plays a crucial role in CNS development. After treatment with resveratrol, CXCR4 expression was markedly upregulated [23], suggesting that CXCR4 is a potential target for alleviating or curing PD.

### Lund human mesencephalic (LUHMES) cells

Lund human mesencephalic (LUHMES) cell line is a subclone of the tetracycline-controlled, v-myc-overexpressing human mesencephalic-derived cell line MESC2.10. LUHMES cells have the same immortalized identity as tumor cells that can be permanently subcultured and maintained. The most distinct feature of this cell line is that it can be differentiated into dopaminergic-like neurons, lessening the gap in PD research and inducing numerous PD-associated phenotypes. Differentiated LUHMES cells exhibit electrical activity, secrete dopamine, and express α-synuclein, in addition to expressing markers of mature dopamine neurons. Moderate expression of dopaminergic specific markers provides faithful data in disease modeling and drug evaluation [25].

The favorable attributes of LUHMES were efficiently used in a study reporting the effect of mutant α-synuclein on dopamine homeostasis [48]. Winner et al. used LUHMES to reveal the toxic property of α-synuclein oligomers [24]. The similar phenotypes of LUHMES and primary neurons under MPTP stimulation confirm the feasibility of mimicking PD with LUHMES [25]. Treatment with 6-OHDA markedly changes the expression of PD-related gene in LUHMES, thereby downregulating the expression of survival genes [26]. This finding suggests that treatment with 6-OHDA weakened mitochondrial function and ion transport, which is consistent with the results for other models treated with 6-OHDA. However, the metabolized effect of 6-OHDA in LUHMES requires further research, and the suitability of 6-OHDA for modeling PD in LUHMES has to be verified.

### MEs23.5

MEs23.5 is a fusion of the neuroblastoma-glioblastoma cell line N18TG2 and rat mesencephalic cells [49]. This cell line acquired immortalized identity from NT18G2 as well as dopaminergic characterization of substantia nigra origin. Meanwhile, this cell line moderately expresses tyrosine hydroxylase and voltage-gated Ca^2+^ channels and can be cultured for a year with a stable phenotype and karyotype. MPTP, 6-OHDA, and rotenone used to treat MEs 23.5 are widely applied to induce cellular phenotypes for drug evaluation. These toxic models have contributed to the identification of potential drugs such as Nesfatin-1 [28], dihydromyricetin [27], and ginsenoside Rg1 [29].

### MN9D

Similar to MEs23.5, MN9D is a hybrid of N18TG2 and rostral mesencephalic tegmentum [50]. MN9D not only expresses TH but also synthetizes, releases, and uptakes dopamine independently [51]. Moreover, MN9D cells can differentiate into dopaminergic (DA) neuron-like cells. Rick et al. indicated that differentiated dopaminergic MN9D cells fail to recapitulate all electrophysiological properties of midbrain dopaminergic (mDA) neurons [52]. Recent comparison of MN9D cells and SH-SY5Y indicates that MN9D cells cultured under normal conditions store high levels of DA but do not convert DA to NE [53]. In contrast to undifferentiated SH-SY5Y cells, MN9D cells exhibit catecholamine uptake characteristics. Therefore, undifferentiated MN9D cells are not suitable for the study of DA neurons. However, shortage of differentiated MN9D cells can be used as a damage model for mimicking the progressive loss of DA neurons in a mechanistic study.

### Primary mDA neurons culture

More than two decades ago, DA neurons in rat-derived postnatal substantia nigra [54, 55] and DA neurons in rat ventral mesencephala [56] were successfully cultured to examine the survival rate of grafted DA neurons in PD patients, which introduced the primary culture of DA neurons. Owing to their sensitivity to neurotoxins, embryonic primary DA neurons are the most commonly used in a large number of studies. MPTP, rotenone, 6-OHDA, and paraquat are added to cultured primary neurons to induce neuronal damage, which mimics the degeneration of DA neurons in PD patients. Many studies identify oxidative stress and mitochondrial defects of DA neurons in PD patients as the effects of neurotoxins [57-59]. However, a comprehensive assessment revealed that these four toxins-induced inhibition of mitochondrial complex I in primary DA neurons derived from Ndufs4 KO mice is not necessary for DA neuronal death [60]. However, the finding that primary cultured neurons alone with a gradient concentration of rotenone seem less damaged than those presented with microglial cells remains inconclusive [61, 62]. Notably, the choice of embryonic or postnatal primary DA neurons in preclinical studies influences research repeatability and credibility. Numerous toxins exert no effect on the midbrain, except for the substantia nigra; thus, the use of embryonic primary DA neurons can dilute the measurement of cell death [63]. The diverse reactions of mice and rats to neurotoxins have also impeded research. Mice are more sensitive to dopaminergic neurotoxins than are rats [64, 65]. Despite uncertainties in the pathogenic mechanisms of neurotoxin-induced neuronal models, they are still the most widely recognized in PD disease modeling for obtaining a rapid and stable disease phenotype.

### Human pluripotent stem cells

Although humans and rodent species are highly homologous in genome, rodent species are still unable to completely mimic disease progression in patients. Modeling PD with human PSCs has been proposed for many years [66]. Theoretically, PSCs can undergo self-renewal and differentiate into any particular somatic cells, providing access to differentiation of DA neurons for PD research [67]. PSCs also enable a more precise genetic profile to identify variations in molecules in PSC-derived somatic cells, given that PSCs can differentiate as described in the embryonic development [68-70]. Modeling PD with PSC-derived specific cell types elucidate the disease. Generally, PSCs can be mainly classified into embryonic stem cells (ESCs) and induced pluripotent stem cells (iPSCs). This section summarizes the application of these two types of PSCs in PD research.

### 1. Modeling PD by using human ESCs

Human ESCs are derived from the inner cell mass of a blastocyst. They can differentiate into all cell types in the body, including DA neurons, which aid in understanding the mechanisms underlying the development of PD [71]. Current *in vitro* differentiation approaches include embryoid body (EB) formation, neural progenitors cell (NPC) differentiation, and floor plate (FP) strategies [72, 73]. EB formation could be efficiently patterned to medial ganglionic eminence and lateral ganglionic eminence [74, 75], while monolayer adherent FP or NPCs could efficiently generate DA neurons displaying the phenotypes of PD [76, 77]. Chi et al. indicated that both EB and adherent culture approaches are highly efficient and allow for anterior neural fates in the absence of exogenous morphogens [78]. To generate appropriate human-derived DA neurons for *in vitro* PD modeling, SHH and FGF8 are the determinants in the differentiation of DA neurons, which act as navigators in cell patterning. The FGF8 signal enhances the early action of striatal neurogenesis along the anterior-posterior axis by triggering the expression of Fgf8, Wnt1, En1, Pax2, and Pax5 but not that of En2 [79]. Meanwhile, FGF8 also plays an essential role in the induction of rostral forebrain DA neurons (A8). Co-treatment with SHH can help restrict the territory from which endogenous DA neurons originate via the dorsal-ventral axis. Yang et al. suggested that exposure to SHH and FGF8 in the early stages could enhance the survival of midbrain neuroepithelial cells when co-transplanted into SNpc, significantly improving the locomotive function of 6-OHDA injured rats [80]. All these studies demonstrate that both SHH and FGF8 are important factors in the differentiation of DA neurons.

Using DA neurons alone is a potential tool for PD research but does not fully recapitulate the brain microenvironment in PD. Post-mortem analysis of PD brain tissues reveals that astrocytes and microglia are involved in the disease process. Astrocytes are neurotrophic and structurally support the normal brain, and microglia provide alerts for brain immune response. In a PD-pathogenic environment, these two groups of cells function abnormally and then hasten the development of PD [81]. On the basis of this finding, a glia-neuron co-culture system is established to explore the progression of PD in the context of glia-neuron interactions. The ESC-derived astrocyte-DA neuron co-culture system is extensively studied. Gunhanlar et al. were the first to report that the astrocyte-DA neuron co-culture system could largely improve mature neurons [82]. Du et al. observed that astrocytes exerted a protective effect on DA neurons [83]. Abnormally functioning astrocytes can accelerate the development of PD to establish activated astrocytes, inducing iPSC-based PD models [84, 85]. However, no studies on the ESC-derived microglia and DA neuron co-culture system have been reported. In summary, these studies emphasize the importance of astrocytes in PD modeling and show that ESCs can be used for drug discovery in PD as well as cell replacement therapy assessment.

Pathological loss of DA neurons, which leads to dopamine reduction, is the primary cause of locomotive defects. Direct differentiation of ESCs into DA precursors for transplantation is a promising technique. Kriks et al. successfully engrafted DA neurons derived from ESCs in 6-OHDA lesion animal models [86], indicating the feasibility of cell therapy for PD. Grealish et al. compared ESC-DA and fetal midbrain DA neurons for implantation in rats [87]. They performed a comprehensive preclinical assessment of ESC-DA neurons, and the results indicated that the functional potency of ESC-DA neurons was similar to that of human fetal midbrain DA neurons. ESC-DA neurons generated using current protocols can be grafted to animal models to restore DA neurotransmission and provide functional benefits [88, 89].

### 2. Modeling PD by using human iPSCs

With the proposal of four specific genes (OCT4, SOX2, KLF4, and c-MYC) or encoding transcription factors in 2006, Shinya Yamanaka pioneered reprogramming technology. Since then, various iPS cell lines have been established by converting somatic cells into pluripotent stem cells [90]. The established iPSCs demonstrates the plasticity of cell fate [91]. Various somatic cells, including blood cells, skin cells, and urinal cells, have been reported to be the cell sources from which iPSCs are obtained. Human iPSCs have opened a promising avenue to study PD owing to their availability and lack of potential ethical concerns associated with human ESCs. The recent development of modeling PD by using iPSCs provides a rich source of cell types that were previously unobtainable and shows potential for elucidating the etiology of PD and developing therapies. Patient-specific and gene-modified iPSCs are two widely used iPS cell types for modeling PD.

Soldner modified the protocol for generating iPSCs free of viral reprogramming factors and with high efficiency of differentiation to DA neurons [92]. Since then, many iPSCs derived from patients with genetic PD were established. Somatic cells from patients with α-synuclein (SNCA), Leucine-rich repeat kinase 2 (LRRK2), PTEN-induced kinase 1 (PINK1), Parkin (PRKN), Deglycase (DJ-1), and ATPase cation transporting 13A2 (ATP13A2) mutations, as well as at-risk individuals carrying β-glucocerebrosidase (GBA) mutations, have thus far been successfully reprogrammed to iPSCs and subsequently differentiated into DA neurons [93]. Disease phenotypes of iPSC-based models focus on the accumulation of alpha-synuclein, autophagic impairment, synaptic defects, and mitochondrial dysfunction. Reprogrammed cells are reset to a naïve state [94], which may hurdle the manifestation of iPSC-based PD models. Overexpression of progerin to induce aging in iPSC-derived DA neurons from patients with PD promotes PD disease phenotypes [94]. Hydroxyurea-treated DA neurons from patients with sporadic PD can rapidly produce PD-related manifestations in early stages [95]. These strategies facilitate the manifestation of disease-relevant phenotypes in a PD model based on iPSCs derived from patients.

Numerous iPSC lines from PD patients have been established with genome editing. Zinc-finger nucleases (ZFNs), transcription activator-like effector nucleases, and the CRISPR/Cas9 system are commonly used tools in genome-editing iPSCs. Genetic correction in A53T (G209A) α-synuclein mutation in PD patient-derived iPSCs was first performed by ZFN-based gene editing [96]. An LRRK2 mutation in PD patient-derived iPSCs was successfully corrected using ZFN-based gene editing and Cre/LoxP systems [97]. LRRK2 G2019S iPSC-derived DA neurons showed mitochondrial DNA damage, which was no longer detected in ZFN-mediated genomic corrected iPSC-derived neurons [98]. By using CRISPR/Cas9 and the piggyBac system, iPSCs with heterozygous LRRK2 G2019S mutation was successfully corrected, leading to a significant decrease in the number of tyrosine hydroxylase-positive neurons and their neurite complexity [99]. The combination of CRISPR/Cas9 and fluorescence-activated cell sorting analysis led to the precise introduction of heterozygous missense A30P and A53T mutations in the SNCA gene into healthy iPSCs. These edited mutant iPSC-derived neuroepithelial stem cells displayed significant defects in nonmitochondrial respiration for extracellular energy flux maximal respiration and ATP production [100].

Human iPSCs provide a powerful platform to model PD for the study of the pathogenesis of PD. They harbor the genetic background of PD patients, presenting the wealth of information that cannot be gained from other available model systems. The use of iPSCs also provides disease-specific drug screening models that are based on patients themselves, which may facilitate the translation of basic science discoveries into clinical treatments. However, efficiently generating functional DA neurons remains challenge in modeling PD by iPSCs. As other cell lines, the iPSCs-based model also fails to mimic locomotive symptoms and even some non-locomotive symptoms, such as autonomic disorders, psychiatric disorders and cognitive decline. Thus, the gap between cell and animal studies should be fully reconciled.

### Organoid culture and Organotypic Culture

Typically, two-dimensional (2D) culture is the most often used approach in disease modeling. However, 2D culture fails to replicate physiologically relevant characteristics like the interaction between glia cells and neurons in a spatially organized microenvironment [101, 102]. To compensate for this deficit, a new advanced *in vitro* disease modeling approach called three-dimensional (3D) culture is developed, which allows for studying functional interactions. Generally, 3D culture is classified into 2 forms: the organoid culture and the organotypic culture. These two types of culture sound the same but actually differ in several aspects.

Organoids are cell-derived and organ-like structures consisting of multiple region-specific cell types. Organoids can be formed by differentiation of ESCs, iPSCs, and progenitor cells of particular organ of interest in 3D cell culture and can also be grown from a limited amount of starting materials coming from tissue biopsies [101, 103]. Both Smits et al. and Kim et al. have developed protocols for human midbrain-specific organoids (hMOs) [101, 103]. In Kim’s study, one healthy control and one LRRK2 G2019S mutant cell line were used to assess the way of organoid forming and disease modeling. Phosphorylated alpha-synuclein (p-S129) was significantly elevated in the LRRK2 G2019S organoid, whereas other PD phenotypes were not apparent, suggesting that neurotoxins are needed to induce PD-related phenotypes [103].

Unlike organoid culture, organotypic culture can only be generated from animal tissues, which is defined the culture of an organ collected from an organism. Daviaud et al. demonstrated that nerve fiber degeneration and striatal dopamine content can be fully characterized and quantified in rat organotypic slice culture [104]. Dal Ben et al. suggested that organotypic slice culture might be stabilized to determine experimental windows [105]. These two studies imply the plasticity of organotypic culture in disease progress. Elfarrash et al. studied inter-neuronal spreading of alpha-synuclein aggregates in rat organotypic slice culture after pre-formed α-syn fibrils (PFF) injection at the dentate gyrus (DG) and found that induced endogenous α-syn aggregates in axons and cell bodies in DG spread to the CA3 and CA1 regions [106]. This model provides evidence on anterograde spreading of the aggregates in PD.

## Animal models of PD

Many comparative studies among a wide range of model organisms, including transgenic animals and invertebrates, contribute to our understanding of the development of PD. Most animal models of PD precisely reproduce the features of the disease, such as the degeneration of DA neurons in SNpc and Lewy body formation with motor dysfunction and several non-motor symptoms [107, 108]. This information provides valuable insights into pathogenic mechanisms and the identification of potential targets. Traditional neurotoxins such as 6-OHDA, MPTP, rotenone, and paraquat/maneb can acutely and rapidly produce a DA-loss phenotype without disease progression. These toxin-based models have considerably helped define the benefits to reduce PD symptoms. Genetic animal models are widely used to study familial PD, however, many transgenic animals with *SNCA*, *LRRK2*, *PINK1*, *DJ-1* and *ATP13A2* mutations cannot sufficiently yield nigral degeneration and typical symptoms [107]. Other transgenic PD models such as the KO mice of SHH, Nurr1, and Atg7 can develop PD-like neuropathology on aging animals. However, no model organisms fully satisfy the human PD criteria. In this section, we provide an intensive analysis of the diversity and similarity of human PD characteristics in various model organisms.

### Drosophila

*Drosophila melanogaster* has been used in the study of various biological processes, such as cell death, proliferation, and migration [109]. *Drosophila* contains a very small number of genes, but it has clusters of DA neurons in the adult brain [110] and exhibits complex behaviors, such as learning and memory, circadian rhythms, and aggression [111]. Thus, the *Drosophila* model has emerged as a particularly effective tool in the study of PD-related genes and PD behavior problems [111]. While other PD models show toxin-based symptoms, the *Drosophila* model shows that feeding with neurotoxin rotenone can cause the degeneration of DA neurons. In addition, exposure of flies to paraquat not only caused the loss of DA neuron clusters but also reduced lifespan as well as PD symptom-like locomotor behavior problems, such as resting tremors, bradykinesia, rotational behaviors, and postural instability. PD symptoms induced by 6-OHDA and MPTP have not been reported in *Drosophila* [112].

*Drosophila* encodes homologs of PD genes, such as *DJ-1*, *PINK1*, *PARKIN*, *LRRK2*, and *VPS35*. Although *Drosophila* PD models cannot fully mimic the features of human PD, loss of DA neurons and locomotor defects have been observed in some PD transgenic flies. Thus, *Drosophila* is suitable for identifying evolutionary conserved pathways and cellular processes associated with PD. Notably, *Drosophila* has no homolog of α-synuclein; however, expression of human wild-type and PD mutant forms of α-synuclein in *Drosophila* not only engenders fibrillar α-synuclein inclusions but also leads to the progressive loss of DA neurons in brain clusters and a reduction in climbing ability. Thus, *Drosophilia* as a model organism is suitable for the study of neurotoxicity induced by α-synuclein and PD pathogenesis mechanisms related to α-synuclein. PINK1 and parkin function in flies are relevant to humans. In their transgenic flies, PINK1 null mutants shared abnormal phenotypic similarities with parkin null mutants, including reduced lifespan, mitochondrial defects, and DA degeneration with locomotor defects [113]. In addition, overexpression of mutants but not wild-type human parkin in flies also led to progressive loss of DA neurons from several clusters, accompanied by progressive locomotor defects. Two DJ-1 orthologs—DJ-1α and DJ-1β—have been identified in *Drosophila* [114-116]. Fly mutants—either with DJ-1α or DJ-1β or with both—exhibit sensitivity to toxins such as paraquat, H_2_O_2_, or rotenone, confirming that DJ-1 protects against oxidative stress. These mutants also showed reduced lifespan and locomotor defects [117]. In *Drosophila*, overexpression of LRRK2 resulted in the loss of DA neurons and locomotor dysfunction [118], with the loss of LRRK2 exerting no effect on the number of DA neurons. However, expression of either WT or mutant forms of human LRRK2 in flies show inconsistent results with respect to PD-related neurodegeneration.

### Caenorhabditis elegans

The nematode *C. elegans* as a model organism is used to explore the mechanisms by which metazoan complexity and basic cellular biology are regulated [119]. *C. elegans* has only 959 cells in the nervous system, of which 302 are neurons; however, they have a dopaminergic circuitry of eight anatomically defined neurons and a well characterized neuronal network [120]. Thus, *C. elegans* is used to provide a basis for understanding PD-associated mechanisms by functional genomic analysis and exploration of previously unknown genetic and environmental risk factors. The neurotoxin 6-OHDA is widely used in *C. elegans*, causing selective DA neuron-related degeneration [121]. Dopamine receptor modulation, autophagy inactivation, an increase in reactive oxygen species (ROS), mitochondrial disruption, and ER chaperones are involved in 6-OHDA toxicity in *C. elegans* [122-124]. Other neurotoxins such as rotenone, MPTP, and paraquat are also used to model neurodegeneration for high-throughput drug screening to identify neuroprotective compounds to reduce cellular death [125].

The *C. elegans* genome encodes genetic homologs to most familial PD genes. However, similar to zebrafish and *Drosophila*, *C. elegan*s does not have a homolog of α-synuclein. Mutation or multiplication of the α-synuclein locus is a widely known cause of PD [126]. Several studies indicate that overexpression of human a-synuclein in *C. elegans* induces progressive time-dependent neurodegeneration and motor behavior defect [127]. Overexpression of LRRK2 induces neurodegeneration in *C. elegans* via overactive kinase activity, whereas blocking of LRRK2 activity could exhibit a disease-modifying effect [128]. PINK1 and Parkin are crucial regulators of mitochondrial autophagy [129]. Their mutations affect mitochondrial morphology, mitochondrial turnover, and mitochondrial biogenesis in *C. elegans* [130]. In addition, overexpression of α-synuclein causes mitochondrial fragmentation, and this mitochondrial damage is rescued by co-expression of wild-type PINK1, parkin, or DJ-1 but not of mutant PINK1, parkin, and DJ-1 [130]. The mechanisms of PD-associated genes that affect mitophagy *in vivo* are less characterized; thus, *C. elegans* is highly suitable for studying the role of mitophagy in the development of PD.

### Zebrafish

The zebrafish, *Danio rerio*, has been used as an excellent vertebrate model for investigating developmental biology, gene function, and human diseases [131]. Zebrafish and humans markedly vary in scale and size, but zebrafish DA neuron clusters in the ventral diencephalon (vDC) are similar to the ascending midbrain DA neurons in the mammalian nigrostriatal system [132]. In adult zebrafish, systemic administration of MPTP or 6-OHDA induces a transient decrease in dopamine level and impairs locomotor behavior [133]. In zebrafish embryos, MPTP and 6-OHDA cause a significant loss of DA neurons in the vDC [134], which is consistent with the findings in humans. Thus, toxin-induced loss of DA neurons in zebrafish is relevant to the evaluation of potential drugs for human PD.

Several orthologs of PD genes have been identified in zebrafish, including Parkin, PINK1, LRRK2, and DJ-1 [135]. These genes have highly conserved functions, such as survival of DA neurons and motor behavior. Both Parkin and PINK1 play critical roles in mitochondrial function. Mitochondrial stress caused transcriptional upregulation of Parkin in zebrafish as in humans. Parkin knockdown in zebrafish led to the loss of DA neurons in the vDC with increased susceptibility to the PD neurotoxin 1-methyl-4-phenylpyridinium (MPP+); moreover, Parkin deficiencies reduced the activity of mitochondrial respiratory chain complex I but caused no abnormal mitochondrial morphology [136]. PINK1 knockdown did not induce large changes in the number of DA neurons in the vDC of zebrafish, but the DA neuron clusters of PINK1-deficient zebrafish were more sensitive to MPTP [136]. PINK1 knockdown also caused mitochondrial defects, such as reduced generation of cristae and a decrease in the number of mitochondria [131]. In addition, the larvae of a PINK1 mutant zebrafish line with a nonsense mutation in exon 7 led to a loss of DA neurons and a decrease in mitochondrial complex I activity, which findings are similar to those in Parkin-deficient zebrafish [131]. The mitochondrial respiratory chain is also impaired in patients with Parkin and PINK1 mutations. Knockdown of DJ-1 did not cause the loss of DA neurons but increased the sensitivity of stress induced by hydrogen peroxide or the proteasome inhibitor MG132 [137]. Notably, deletion of the WD40 domain of LRRK2 in zebrafish resulted in the loss of DA neurons in the vDC and locomotor defects [138]. To summarize, transgenic zebrafish provide powerful tools to explore the *in vivo* roles of PD genes. However, the ortholog of α-synuclein has yet to be found in zebrafish; therefore, transgenic zebrafish is not a good model for investigating the pathology of α-synuclein.

### Rodents

Rodent PD models present many advantages, including genetic and physiological similarities to humans. Owing to affordability, relatively short life span of rodents, robustness of genetic modulation technologies for rodents, and abundance of references on the physiology and behaviors of rodent, the rodent model is the most widely used model in both the basic study of PD and drug development for PD treatment. These models can be generally divided into two categories: neurotoxic models and transgenic models. With its own advantages and limitations, each model recapitulates one or several pathological features of PD.

### 1. Neurotoxic models

Several toxic agents, including 6-OHDA, MPTP, paraquat, and rotenone have been used to establish rodent PD models over the past decades. A common feature of these toxic models is selective death of dopaminergic neurons, which is the major pathological hallmark of PD. Toxicants regarded as environmental risk factors are widely used to establish sporadic PD. However, these neurotoxins-induced rodent models have several disadvantages. For instance, rapid degeneration of dopaminergic neurons is inconsistent with slow degeneration in PD patients. Moreover, typical PD pathological markers—that is, intraneuronal inclusions—are not found in most toxin-induced models.

The neurotoxic synthetic organic compound 6-OHDA exhibits a structure similar to that of DA neurotransmitters. These neurotransmitters have high affinity for DATs and thus gain access to the cytosol where they can auto-oxidize, generating intracellular oxidative stress [139]. This compound cannot cross the blood-brain barrier, requiring direct injection into the SNpc or the striatum. 6-OHDA is frequently injected unilaterally because bilateral injection of this compound into the striatum leads to severe adipsia, aphagia, and even death [140]. Similar to many other neurotoxic PD models, 6-OHDA causes acute neurodegeneration without the progressive, age-dependent properties of PD. In addition, Lewy bodies or α-synuclein-positive aggregates do not exist in this model.

MPTP is converted into MPP+ to produce neurotoxicity, which selectively enters dopaminergic neurons via DAT and inhibits mitochondrial respiratory complex I [141]. NHPs such as monkeys are highly sensitive to MPTP, replicating almost all hallmarks of PD, including oxidative stress, reactive oxygen species, energy failure, and inflammation. Mice are less sensitive to MPTP, and rats are resistant to this toxicity [142]. The MPTP-induced PD mouse model is one of the most commonly used animal models for analyzing the effect of drugs that act on dopaminergic neurons [143]. Fornai et al. found that continuous low-level exposure of mice to MPTP caused the formation of inclusion bodies in remaining SNpc neurons, although no α-synuclein-positive aggregates were observed in most cases [144].

Rotenone is a widely used pesticide that acts as a mitochondrial complex I inhibitor. Chronic exposure to low doses of rotenone leads to inhibition of the mitochondrial electron transport chain in the brain. In a rotenone-induced rat PD model, the mitochondrial respiratory function and nuclear transcription factor 2 in the substantia nigra were significantly impaired[145]. Similar to MPTP, rotenone is highly lipophilic, readily crosses the blood-brain barrier, and induces most hallmarks of PD, including α-synuclein aggregation and Lewy-like body formation [146, 147]. Rotenone leads to significant loss of TH-positive neurons and behavioral impairment. In addition, α-synuclein immunoreactivity increased in surviving TH-positive neurons in rotenone-treated mice [148].

Paraquat is an herbicide that inhibits photosynthesis, affects ROS formation, and causes oxidative stress. It was first considered as a possible toxin inducer of PD in the mid-1980s because of the similarity of its chemical structure to that of MPP+ [149]. Unlike MPP+, paraquat can penetrate the blood-brain barrier independent of age and species, with young mice (<8 weeks) being more susceptible to paraquat penetration into the brain [150]. Systemic administration of paraquat in mice induces loss of dopaminergic neurons and motor deficits independent of dose [151, 152] and age [152, 153]. Paraquat can also cause upregulation and aggregation of α-synuclein and form LB-like structures in individual DA neurons in SNpc [154]. Paraquat-induced PD can improve by adding maneb (another neurotoxic herbicide) [155]. These models support the theory that environmental pesticides contribute to the development of PD [150, 156, 157].

Acrolein is a toxin exogenously caused by environmental pollution [158] and endogenously produced by lipid peroxidation of polyunsaturated fatty acids, DNAs, and proteins as well as by metabolism of allyl compounds [159]. Acrolein can sufficiently induce neurodegeneration of the nigrostriatal dopaminergic system in rats. Oxidative stress is considered as the main mechanism of acrolein-induced cytotoxicity. The model simulates neurodegeneration, oxidative stress and protein accumulation, neuroinflammation, and programmed cell death in the nigrostriatal dopaminergic system. In addition, apomorphine-induced turning behavior was evident in rats subjected to unilateral infusion of acrolein in substantia nigra [160].

### 2. Genetic models

In addition to the aforementioned toxin-induced models, transgenic rodent models also bear considerable significance for PD research. In accordance with the discovery of genes related to familial PD, transgenic models are established by introducing or depleting these PD-related genes in animals. Twenty-three PARK genes have thus far been associated with PD. Mutations in at least 5 genes (SNCA, PRKN, PINK1, PARK7 and LRRK2) among these PARK genes are directly linked to the pathogenesis of PD. Some genes have not been conclusively proved (EIF4G1, GIGYF2, HTRA2, PARK3, PARK5, PARK10, and PARK12), while others are considered as risk factors (BST1, GAK, GBA, HLA, MAPT, and PARK16) [161, 162]. Reports suggest that mutations in ATP13A2, F-Box Protein 7 (FBXO7), and Phospholipase A2 Group (PLA2G) cause early-onset L-dopa-responsive Parkinsonism with pyramidal signs [163]. More variants have been found to be associated with PD, but most have yet to be replicated in independent studies. The development of genetic animal models offers an opportunity not only to study the pathogenesis of PD but also to evaluate new treatments that focus on modifying the disease rather than just alleviating the symptoms [164]. In the current study, we reviewed well-established transgenic PD rodent models based either on autosomal dominant inheritance (such as SCNA, LRRK2, and UCHL1) or autosomal recessive inheritance (such as PRKN, PINK1, and DJ-1) related genes.

**Table 2:** Key features of common models of PD in rodents.

Models	Mechanism	DA neuron loss	Behavioral symptoms	α-Syn aggregation	Disadvantages
6-OHDA induced [176]	Oxidative stress	Yes (Acute)	Rotational behavior after unilateral injection	No	Acute loss of DA neurons; Require intracerebral injection; Very little synuclein involvement
MPP+ induced [176]	Inhibition of the mitochondrial electron transport chain.	Yes (Acute)	Less obvious motor impairments in acute rodent models	No	Acute loss of DA neurons; Inclusions are rare
Rotenone induced [177]	Inhibition of the mitochondrial electron transport chain	Yes	Decreased motor activity	Yes	Substantial morbidity and mortality; Labor and time intensive
Paraquat induced [178]	Oxidative stress	Yes	Decreased motor activity	Yes	Severe systemic toxicity.
Acrolein induced [160]	Oxidative stress	Yes (Acute)	Turning behavior was evident after unilateral injection	Yes	Recently reported with little publication; Require intracerebral injection
α-synuclein (Overexpressing) [173]	α-syn toxicity	Mild or no.	Decreased motor activity	Yes	No obvious DA neuron death observed with α-synuclein models
α-synuclein (rAAV injection) [179]	α-syn toxicity	Yes	Decreased motor activity	Yes	Complex operations
α-synuclein (PFF inoculation) [179]	α-syn toxicity.	Yes	Decreased motor activity	Yes	Complex operations
UCH-L1 or Psmc1 (KO or mutant) [180, 181]	UPS dysfunctions	Yes.	Decreased motor activity	Yes	Limitation in the study of special gene related familiar PD
Parkin (KO or mutant) [182]	Parkin depletion	Mild or no	No major olfactory, emotional or motor impairments	No	General lack of degeneration, motor impairments and α-synuclein aggregation
PINK1 or DJ-1 (KO or mutant) [183]	Mitochondrial dysfunctions	Mild or no	Decreased motor activity	No	General lack of degeneration and α-synuclein aggregation
LRRK2 (KO or mutant) [184]	LRRK2 depletion	Mild or no	Decreased motor activity	No	General lack of degeneration and α-synuclein aggregation

## SNCA (PARK1/PARK4)

The misfolded protein referred to as α-synuclein is the main component of Lewy bodies in the brain of PD patients. The encoding gene SNCA was first found to be associated with familial PD, and several α-synuclein missense mutations have been identified, including A53T, A30P, E46K, G51D, and H50Q [165]. In addition, dual or triple copies of SNCA (PARK4) were sufficient to cause PD, indicating that α-synuclein expression is a key factor in PD development [126, 166].Many rodent lines overexpressing α-synuclein or its mutation have been generated in recent decades. Notably, common polymorphisms of the SNCA promoter affects the risk of developing idiopathic PD by influencing α-synuclein expression. Under the neuron-specific platelet-derived growth factor β (PDGFβ) promoter, transgenic mice overexpressing WT human α-synuclein exhibit motility disorders and dopaminergic terminal loss in the basal ganglia, as well as α-synuclein and ubiquitin-immunoreactive inclusions in cortical, hippocampal, and nigral neurons [167]. Under the control of mThy1 regulatory sequences, mice overexpressing WT or mutant human α-synuclein replicated most PD characteristics, including progressive α-synuclein pathology, neuronal degeneration, and motor deficits [168]. The expression of a-synuclein in mice induced by the mThy1-1 gene is higher than that by PDGF, and highly expressed α-synuclein is distributed throughout the brain, including the basal ganglia and thalamus [169]. However, van der Putten et al. reported that α-synuclein pathology was absent in substantia nigra in mThy1-h [A53T] α-synuclein transgenic mice [170]; moreover, in another mThy1- h [A30P] α-synuclein transgenic mouse model, proteinase K-resistant α-synuclein was absent in the striatum and substantia nigra [171, 172]. These results suggested α-synuclein pathology could be different between different mouse models. Under the control of the mouse prion promoter, transgenic mice expressing A53T but not WT a-synuclein exhibited delayed-onset motor deficit, accompanied by the accumulation of toxic filamentous a-synuclein cytoplasmic inclusions throughout the neuroaxis, recapitulating features of human counterparts [173]. These mice developed neuronal mitochondrial degeneration concomitant with the development of α-synuclein aggregates and caused motor neuron loss (approximately 75%) [174]. In rats, the BAC-driven expression of E46K α-synuclein lacks dopaminergic neurodegeneration but exhibits α-synuclein aggregation, altered dopaminergic metabolism in the striatum, and oxidative stress damage [175]. Owing to the significance of α-synuclein in PD pathology, numerous models have been developed with either the WT or disease-associated mutant protein, in addition to the previously mentioned models (Table 2).

Stereotactic injection of recombinant adeno-associated virus (rAAV) expressing α-synuclein and preformed α-synuclein fibrils (PFFs) are two other methods of modeling α-synuclein pathology in rodents. Overexpression of human WT α-synuclein or PD-associated mutants, such as A53T, A30P, S129A, and S129D α-synuclein, have triggered characteristics associated with PD, including dopamine neuron loss, reduced dopamine content, motor impairments, and neuroinflammation [179]. rAAV-α-synuclein can be injected into rat or mouse brains to induce the PD models, providing a choice to test whether certain genes affect neurodegeneration synergistically with α-synuclein [185]. The PFF model is established by injecting α-synuclein fibrils into the brain to act as a seed to recruit endogenous α-synuclein, leading to the formation of larger aggregates that exert toxic effects on the affected neurons [186, 187]. This model produces Lewy body-like α-synuclein fibrillar inclusions in a process most similar to PD in humans. Significant neurodegenerative changes in midbrain DA neurons can take up to six months when PFF is injected into the striatum or substantia nigra [186, 188, 189]. The advantages of rAAV- and PFFs-mediated models are (i) a large number of α-synuclein transgenic rodents need not hybridize with other transgenic rodents of interest when studying the influence of different genes on PD-related pathology and phenotype and (ii) the models can be more quickly established compared with other genetic models.

## PRKN (PARK2)

Mutations of PRKN are the most common cause of early-onset and juvenile PD [190]. Parkin is an E3 ubiquitin ligase involved in protein ubiquitination. When combined with PINK1, parkin plays important roles in mitophagy and ROS scavenging, participating in the quality control of mitochondria [191]. Several parkin KO mice (exon 2, exon 3, or exon 7) have been developed; however, these mouse models display only a mild or no phenotype without loss of DA neurons [182, 192, 193]. The parkin KO (EX2) mouse model is not a robust model of PD. For up to 22 months, no evidence of disruption of the nigrostriatal pathway, cognition, or noradrenergic system was observed [193]. Parkin KO (EX3) mice showed behavioral paradigm defects sensitive to nigrostriatal impairment, mitochondrial dysfunction, and oxidative damage but no dopaminergic neuron loss [182]. Lack of parkin (EX3) recently anticipated the phenotype and affected mitochondrial DNA levels and morphology in PD-mito-PstI mice [194]. By deletion of parkin exon 7, the transgenic mice exhibit catecholaminergic neuron loss in the locus coeruleus. Norepinephrine is significantly reduced in discrete regions of the central nervous system, resulting in significantly reduced norepinephrine-dependent startle response. However, similar to exon 3-deficient mice, no damage to the nigrostriatal DA neurons was found [192]. Meanwhile, transgenic mice that selectively overexpressed the C-terminal truncated human mutant parkin (Parkin-Q311X) in DA neurons showed age-dependent hypokinetic motor deficits, dopaminergic neuron degeneration, and accumulation of proteinase K-resistant α-synuclein [195, 196]. Studies on Parkin-Q311X mice elucidate the mechanism underlying the toxicity of parkin. In addition, age-related changes in oxidative stress play an important role in the etiology of PD, which may be reflected in Parkin-Q311X mice.

## PINK1 (PARK6) and DJ-1 (PARK7)

PINK1 mutations are the second most common cause of autosomal recessive early-onset PD [197]. Evidence indicates that the PINK1 protein protects against neuronal death induced by mitochondrial dysfunction and deficits in clearance systems. PINK1-deficient mice showed impaired striatal dopamine release and synaptic plasticity, progressive weight loss, and reduced spontaneous motor activity. However, similar to parkin-deficient mice, most PINK1 KO mice exhibited reduced striatal DA levels without neurodegeneration [198, 199]. PINK1-deficient mice have also exhibited extensive mitochondrial dysfunction and increased oxidative stress [200]. Loss of PINK1 significantly increased the damage caused by systemic MPTP treatment, providing strong evidence for the role of endogenous PINK1 in maintaining neuronal survival [201].

DJ-1 is a widely expressed and highly conserved dimeric protein. It is involved in many cellular processes and has a major function in neuronal protection against oxidative stress. Mice deficient in DJ-1 caused increased oxidative stress, visual dysfunction, retinal abnormalities, and progressive behavioral deficits but not loss of DA neurons [202-205]. Combinations of DJ-1 transgenic and MPTP models increased the sensitivity of DJ-1 mutant mice to MPTP. Restoring DJ-1 expression in DJ-1 KO mice can improve all phenotypes. These results demonstrated that DJ-1 protects neurons from oxidative stress, whereas DJ-1 deficiency may lead to a hypersensitivity response of dopaminergic damage, causing PD [206].

## UCH-L1 (PARK5)

The pathogenic effects of UCH-L1 mutations have been debated owing to their rarity in PD patients [207, 208]. Ile93Met mutation in UCHL1 was first identified in a German family with PD, resulting in decreased ubiquitin hydrolase activity, abnormal proteolytic pathways, and protein aggregation [209]. UCHL1 comprises 1%-2% of the total brain soluble protein, and a lack of UCHL1 expression in mice can lead to the gracile axonal dystrophy (*gad*) phenotype. However, the pathological and clinical features of *gad* mice are inconsistent with those of PD [210]. Setsuie et al. generated Ile93Met-overexpressing mice and observed an age-dependent degeneration of TH-positive DA neurons in the substantia nigra. Although no Lewy body formation was observed, silver-stained positive particles and abnormal electron dense-nucleoli occurred in degenerating DA neurons. In addition, mutant UCH-L1 exacerbates pathology in MPTP-treated transgenic mouse models [180]. Yasuda et al. overexpressed α-synuclein in Ile93Met transgenic mice and *gad* mice. Yasuda et al. observed a significantly enhanced loss of DA neurons in Ile93Met transgenic mice rather than *gad* mice [211]. These results suggest that pathogenicity of PARK5 is attributed to the acquired toxic function of the UCH-L1Ile93Met mutant.

## LRRK2 (PARK8)

LRRK2 is a large protein (2,527 residues) consisting of a kinase domain, a guanosine triphosphatase domain (ROC-COR domain), and other motifs [212]. G2019S in kinase domain and R1441G in GTP domain are the most common mutations associated with PD, leading to an abnormal increase in LRRK kinase activity that affects various cellular processes, including vesicle transport and immune response [213]. Highly selective LRRK2 inhibitors have been reported, but the effectiveness of the inhibitors has yet to be verified in PD patients.

Several transgenic techniques have been applied to establish pathological models of LRRK2 in rodents, including tetracycline transactivator-controlled inducible transgenic [214], CMVE/PDGF transgenic [215], Thy1 transgenic [216], BAC transgenic [217-221], and knock-in techniques [222, 223]. However, most models do not have or mildly replicate PD phenotypes. In mice with LRRK2 G2019S mutants, only decreased DA content and release in the striatum were observed with age [217]. Specifically, in an LRRK2 R1441G mutant transgenic mouse model, progressive motor defects were observed at the age of 10-12 months, resulting in almost complete immobility; however, the number of DA neurons remained unchanged [224]. Both BAC-LRRK2 R1441G and G2019S transgenic rats showed no signs of neurodegeneration or significant cognitive or motor deficits with age. In LRRK2 G2019S rats, conflicting results have been reported on the rotarod test: transgenic animals performing better than WT animals in some studies but significantly worse in others (3-6 months) [218, 225]. In addition, BAC-controlled expression of G2019S LRRK2 induced oxidative stress in striatum and substantia nigra, increasing iNOS expression [220]. Although the phenotypes of LRRK2 transgenic rodent models are mild and not typical PD-like, a combination of transgenic models can enhance PD-like pathology. Overexpressing LRRK2 in A53T α-synuclein transgenic mice promoted dopaminergic degeneration and α-synuclein aggregation [214, 226]. Main rodent models are listed in Table 1 to provide a better comparison of these animal models.

With the difficulties related to ethical approval and high gene homology to humans considered, rodents are often the first choice for disease modeling. In nature, PD is specific to humans; thus, rodents need to be exposed to multiple environmental toxins (i.e., 6-OHDA, MPTP, rotenone, and paraquat) to induce PD pathologies. Well-established rodent animal models can mimic the pathologies of PD, including loss of DA neurons, accumulation of overproductions of abnormal proteins, and locomotive defects. However, physiological concerns regarding rodent models still arise. One reason is that rodent animals exhibit drug resistance to MPTP to which humans are susceptible. Another reason is that differences in blood-brain barrier exist between rodents and humans. Regardless, rodents are the most important resource for the study of PD, and these limitations should just be considered in the analysis.

### Domestic Pigs

Pigs have the advantages for mimicking human disease due to their unique features exhibiting not only high homology of human genes but also similarities in anatomy, metabolism, neurobiology and physiology to humans. Yet surprisingly, current technologies are still unable to generate porcine ESCs for gene editing. Somatic cell nuclear transfer (SCNT) technology, which was first used to clone the sheep dolly, has been employed to generate genetic PD pigs [227-230]. Zhou et al. applied Cas9/sgRNAs to obtain heterozygous mutant porcine fetal fibroblasts to serve as nucleus donors and further generated homozygous gene-targeted pigs through a single round of SCNT in that 15 tyrosinase (*TYR*) biallelic mutant pigs (TYR^-/-^) and 20 *PARK2* and *PINK1* double-gene KO pigs (PARK2^-/-^, PINK1^-/-^) were successfully generated without detecting any off-targets [229]. Yao el al. combined TALENs technology with SCNT to efficiently generate bi-allelic KO pigs [229]. Despite there were no integration of exogenous DNA and determination of off-targets, the low success ratio raises the cost concern in generation of familial PD pigs. Both Zhou’s and Yao’s studies reported that mutant pigs exhibit some gene-related function impairment with only mild PD-related symptoms and phenotypes. The long-life span of pigs may contribute to the difficulties in phenotypic manifestation, as PD is an age-related disease. Notably, current studies show that nearly 70% cloned pigs possess normal telomere, however, accumulation of abnormal macromolecules, incomplete reprogramming, and long-life expectancy may reduce the confidence in efficiently generating genetic PD pigs by SCNT [231]. One alternative and novel approach to generate biallelic knockout pigs in one step is direct cytoplasmic injection of Cas9 mRNA and sgRNA into porcine zygotes [227], which may provide evidence on efficiently generating large genetic animals to study PD.

### Nonhuman primates

NHPs are closely homologous to humans with respect to monoaminergic transporters, DA distribution and metabolism, cognitive behaviors, and developmental and aging processes [232-234]. The NHP model provides a useful platform for understanding PD pathology and evaluating PD therapy [140, 235]. Owing to its ability to bypass the blood-brain barrier and its metabolite, MPTP is commonly used to induce Parkinsonism in NHP. The MPP+ ion can be selectively taken up by DA neurons via DATs and further damage the DA neurons in the substantia nigra, which finding is similar to that observed in human PD [236]. In addition, MPTP-treated monkeys can develop stable and bilateral clinical features such as rigidity, postural tremor, eyelid closure, and many other symptoms closely mimicking human PD [237, 238]. These motor behavior defects could be relieved by supplementation with L-Dopa, as exactly observed in PD patients [237]. Human PD-like dyskinesia in MPTP-treated monkeys can be used to investigate the efficacy of potential anti-dyskinetic therapies. Non-motor symptoms occur in PD patients before the onset of motor impairment, and some clinical PD symptoms do not result from the depletion of DA neurons. MPTP-treated monkeys also exhibit several non-motor symptoms, such as cognitive deficit, sleep/wake problem and gastrointestinal disturbances [239-241], and Lewy pathology [242], suggesting that MPTP-treated monkey models are the best human-like PD model for the study of pathogenic mechanisms and exploring potential therapies.

Two reports have thus far used the transgenic NHP PD model to investigate the early pathology of PD. Niu et al. generated six transgenic rhesus monkeys with α-synuclein A53T mutation via lentiviral vector expressing A53T in fertilized monkey eggs [243]. Age-dependent non-motor symptoms, such as cognitive defects and anxiety phenotypes, were observed; however, no without sleeping disorders were reported, suggesting that mutant α-synuclein can lead to specific symptoms of early-onset PD in NHPs. The other study by Yang et al. found that α-synuclein was increased in the brain of older monkeys [244]. They further used the stereotaxic injection of lentiviral vectors expressing mutant α-synuclein (A53T) into the substantia nigra of monkeys and found that aging also caused an increase in the accumulation of α-synuclein A53T in neurites. Notably, mutant α-synuclein led to more degeneration and increased the number of reactive astrocytes in the injected substantia nigra in the monkey brain relative to that in the mouse brain, suggesting that the NHP model of PD is more sensitive to the toxicity of α-synuclein mutant.

## Conclusion

PD is one of the most debilitating diseases affecting 1%-2% of the population aged over 65. Selective loss in SNpc and synucleinopathy are the characteristic pathological hallmarks of PD, leading to movement disorders such as rigidity, resting tremor, bradykinesia, and postural instability. Our current knowledge regarding the pathogenic mechanisms of PD is mostly derived from various experimental PD models established in the past decades. Experimental models can represent various aspects of the disease at different levels of cells and/or molecules, movement and non-movement behaviors, and even electrical activity. Each currently existing experimental model has its distinct use but none can fully recapitulate the pathological and/or phenotypic features of PD. Selection of the PD model largely relies on the aspect of the disease being studied and the kind type therapy to develop. In the study of multifactorial and complex neurodegenerative diseases such as PD, dissecting complex pathological processes into simpler molecular events is particularly useful. Cell-based models have reproduced some major features of PD, particularly the comprehensive biochemical pathways such as oxidative stress, mitochondrial impairment, autophagy dysfunction, neuroinflammation, and apoptosis of DA neurons. Thus, cell models can provide distinct opportunities for identifying molecular pathogenesis and large-scale testing of potential compounds. Each cellular model has its own advantages and disadvantages in the molecular pathogenic study and drug testing. Regardless, cell models are evidently unable to recapitulate *in vivo* pathogenesis and pathophysiology requiring the interaction of different cell types [245].

Animal models provide valuable insights into the pathogenic mechanisms of PD. Nonmammalian animal models contribute to the learning of some general phenotypes involved in the development of PD; however, whether the heterogeneity of highly conserved genes can be reproduced in humans remains largely unknown. More importantly, these small animals cannot well replicate clinical manifestations such as the loss of DA neurons. Given the difficulty of ethical identification and the high homology with human genes, rodents are often the first choice for disease modeling. An ideal rodent model for PD displays age-dependent and progressive loss of DA neurons, motor dysfunction, and abnormal α-synuclein pathology. However, both currently existing toxic and transgenic rodent PD models exhibit their own distinct characteristics and limitations. Therefore, the selection of one animal model or another relies on the specific objectives being pursued. Although the pathological symptoms of PD are widely known to result from the selective loss of DA neurons in the nigrostriatal pathway, recent studies suggest that the pathology originates outside the brain [246]. Indeed, α-synuclein pathology originating in the gastrointestinal tract and then transmitted to the brain via the vagus nerve in patients with PD was first reported by Heiko Braak et al. [247] and then recently demonstrated in rodent models by two groups [248, 249]. These reports suggest the role of the gut-brain axis in the initiation and propagation of PD pathology. Another study has recently reported that intestinal infection with Gram-negative bacteria in Pink1^-/-^ mice acts as a triggering event in PD [250]. These new rodent models undoubtedly provide valuable tools to further explore the relevance of the gut-brain axis in the initiation and propagation of PD.
